# Supplementary material for: Maternal intermittent fasting in mice disrupts the intestinal barrier leading to metabolic disorder in adult offspring
Source: Commun Biol. 2023 Jan 12;6:30. doi: 10.1038/s42003-022-04380-y (PMC9834385; doi:10.1038/s42003-022-04380-y)
Supplement: Supplementary file 2 — Supplement [file 42003_2022_4380_MOESM2_ESM.pdf]

Supplementary Figures

Figure S1. Effects of intermittent fasting on maternal metabolism

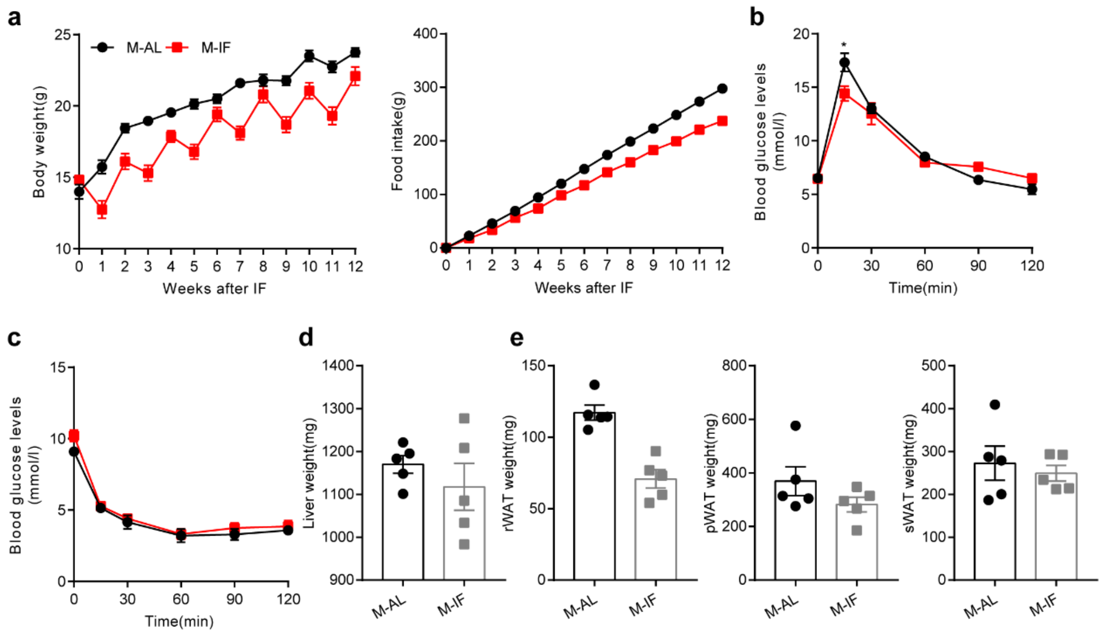

Four-week-old female mice were fed ad libitum or fasted intermittently for 12 weeks. M-AL: mother dam fed ad libitum. M-IF: maternal intermittent fasting. Results were expressed as mean±SEM. $^{*}P<0.05$ vs. M-AL.

(**a**) Body weight and accumulative food intake. N=10.

(**b**) Glucose tolerance test (OGTT).

(**c**) Insulin tolerance test (ITT). OGTT and ITT were carried out at the end of intermittent fasting.

(**d**) Liver weight.

(**e**) Fat mass of rWAT, pWAT and sWAT. (**b-e**) N=5.

Figure S2. Maternal intermittent fasting deteriorates offspring glucose and lipid metabolism

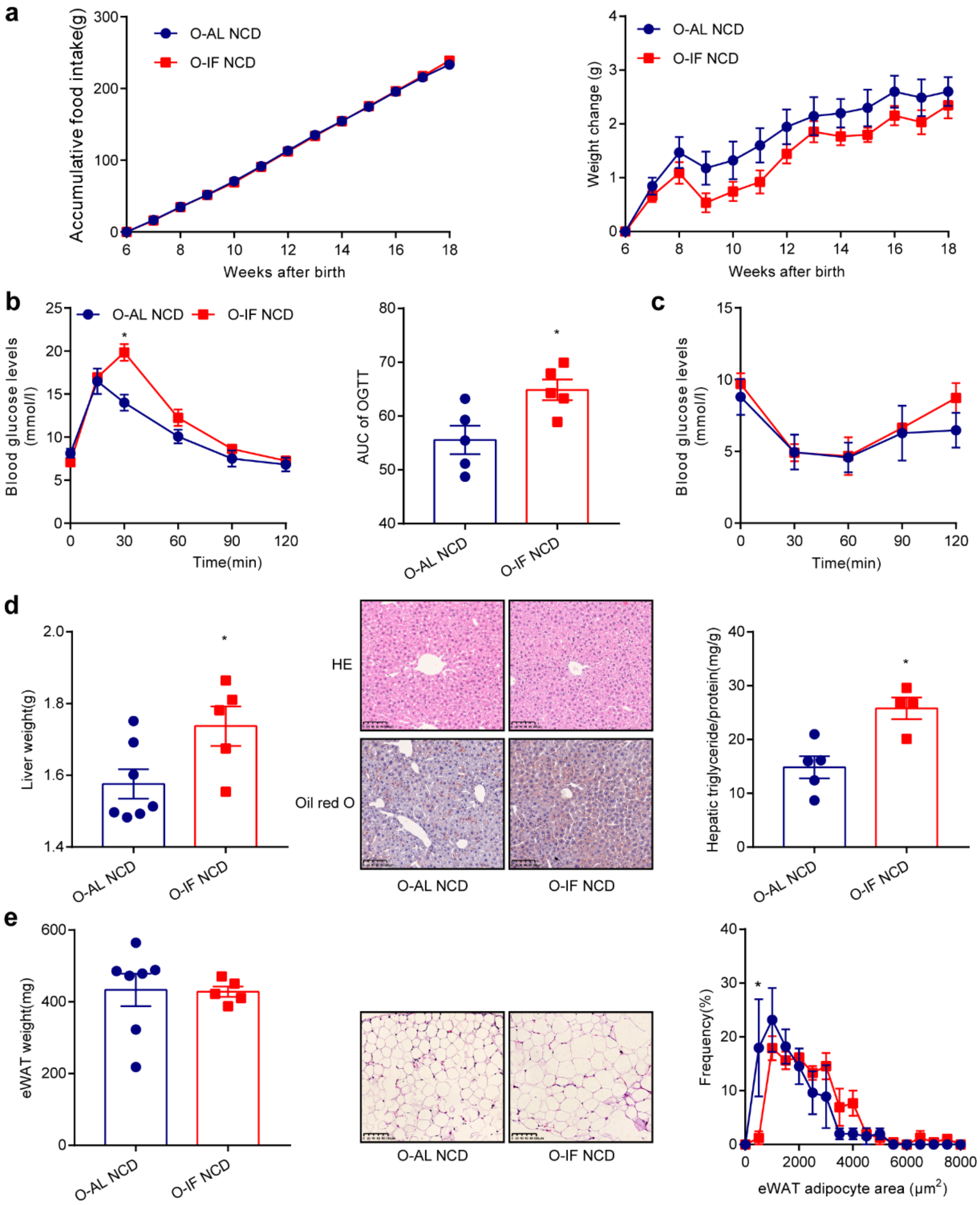

Six-week-old offspring of M-AL group and M-IF group were fed normal chow diet (NCD) for 12 weeks. Results were expressed as mean±SEM. *P <0.05 vs O-AL NCD.

(**a**) Accumulative food intake and changes of body weight. N=9.

(**b**) Glucose tolerance test and the area under curve. N=5.

(**c**) Insulin tolerance test. N=5.

(**d**) Liver weight, steatosis and lipid contents in liver. N=7 and 5 for O-AL NCD and O-IF NCD respectively.

(**e**) Fat mass, H&E staining and adipocyte size of eWAT. N=7 and 5 for O-AL NCD and O-IF NCD respectively.

Figure S3. Intestinal barrier in maternal intermittent fasting offspring fed normal chow diet

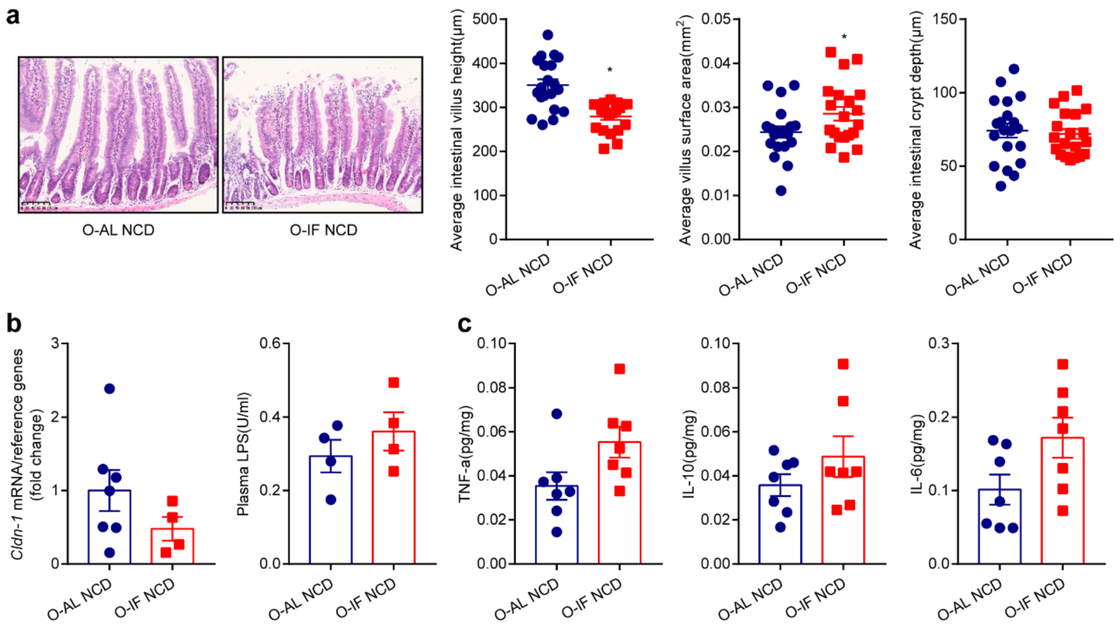

(**a**) Intestinal histomorphology: H&E staining of the small intestine and quantitative results of villus height, villus surface area, and crypt depth.

(**b**) mRNA levels of *cldn-1* and plasma levels of LPS. N=4.

(**c**) Inflammatory cytokines of intestine, measured through AimPlex Multiple Immunoassays for Flow. Results were normalized by total protein level in tissue extracts. Data was expressed as mean ± SEM. *P <0.05 vs O-AL NCD. N=7.
