# Supplementary material for: Maternal intermittent fasting in mice disrupts the intestinal barrier leading to metabolic disorder in adult offspring
Source: Commun Biol. 2023 Jan 12;6:30. doi: 10.1038/s42003-022-04380-y (PMC9834385; doi:10.1038/s42003-022-04380-y)
Supplement: Supplementary file 5 — reporting-summary [file 42003_2022_4380_MOESM5_ESM.pdf]

nature portfolio

Corresponding author(s): Yue Yin and Weizhen Zhang

Last updated by author(s): Nov 15, 2022

Reporting Summary

Nature Portfolio wishes to improve the reproducibility of the work that we publish. This form provides structure for consistency and transparency in reporting. For further information on Nature Portfolio policies, see our Editorial Policies and the Editorial Policy Checklist.

Statistics

For all statistical analyses, confirm that the following items are present in the figure legend, table legend, main text, or Methods section.

n/a	Confirmed	
☐	☒	The exact sample size (*n*) for each experimental group/condition, given as a discrete number and unit of measurement
☐	☒	A statement on whether measurements were taken from distinct samples or whether the same sample was measured repeatedly
☐	☒	The statistical test(s) used AND whether they are one- or two-sided<br>*Only common tests should be described solely by name; describe more complex techniques in the Methods section.*
☐	☒	A description of all covariates tested
☐	☒	A description of any assumptions or corrections, such as tests of normality and adjustment for multiple comparisons
☐	☒	A full description of the statistical parameters including central tendency (e.g. means) or other basic estimates (e.g. regression coefficient) AND variation (e.g. standard deviation) or associated estimates of uncertainty (e.g. confidence intervals)
☒	☐	For null hypothesis testing, the test statistic (e.g. *F*, *t*, *r*) with confidence intervals, effect sizes, degrees of freedom and *P* value noted<br>*Give P values as exact values whenever suitable.*
☒	☐	For Bayesian analysis, information on the choice of priors and Markov chain Monte Carlo settings
☒	☐	For hierarchical and complex designs, identification of the appropriate level for tests and full reporting of outcomes
☒	☐	Estimates of effect sizes (e.g. Cohen's *d*, Pearson's *r*), indicating how they were calculated

Our web collection on statistics for biologists contains articles on many of the points above.

Software and code

Policy information about availability of computer code

Data collection	(1)Intestinal cytokines were assayed in an AimPlex Platform employing mouse Th1/Th2/Th17 18-plex kit (C281118, Beijing QuantoBio Biotechnology Co., Ltd., Beijing, China) following the manufacturer's instructions.<br>(2) Gut microbiota were analyzed by Nonogene Biotechnologies Inc., Beijing using primers that target to the V3-V4 regions of 16S rRNA.<br>(3)The purified RNA was quantified using a NanoDrop spectrophotometer (Agilent Alto, CA, USA).
Data analysis	GraphPad Prism 7, Image J

For manuscripts utilizing custom algorithms or software that are central to the research but not yet described in published literature, software must be made available to editors and reviewers. We strongly encourage code deposition in a community repository (e.g. GitHub). See the Nature Portfolio guidelines for submitting code & software for further information.

Data

Policy information about availability of data

All manuscripts must include a data availability statement. This statement should provide the following information, where applicable:

- Accession codes, unique identifiers, or web links for publicly available datasets
- A description of any restrictions on data availability
- For clinical datasets or third party data, please ensure that the statement adheres to our policy

All data supporting the findings of this study are available within the Article and Supplementary Files or available from the corresponding author upon request. Raw sequences have been deposited on NCBI public repository (Bioproject # PRJNA774493).

Field-specific reporting

Please select the one below that is the best fit for your research. If you are not sure, read the appropriate sections before making your selection.

☒ Life sciences ☐ Behavioural & social sciences ☐ Ecological, evolutionary & environmental sciences

For a reference copy of the document with all sections, see nature.com/documents/nr-reporting-summary-flat.pdf

Life sciences study design

All studies must disclose on these points even when the disclosure is negative.

Sample size	(1)For maternal mice, n=5-10.<br>(2)For offspring mice without treatment, n=4-10.<br>(3)For offspring mice with L.intestinalis, n=3-5.<br>(4)For cohoused-offspring mice, n=4-6.
Data exclusions	PCR data were excluded based on sample degradation, observed by the expression of reference genes.
Replication	Except for 16S rRNA sequencing, other experiments were repeated.
Randomization	Maternal mice were randomly assigned to different groups.
Blinding	Animal treatments were not done blind. However, standard randomization procedures were strictly followed with proper controls included in all experiments. Biological samples, and physiological data were collected and analyzed under the same conditions.

Reporting for specific materials, systems and methods

We require information from authors about some types of materials, experimental systems and methods used in many studies. Here, indicate whether each material, system or method listed is relevant to your study. If you are not sure if a list item applies to your research, read the appropriate section before selecting a response.

Materials & experimental systems

n/a	Involved in the study	
☐	☒	Antibodies
☒	☐	Eukaryotic cell lines
☒	☐	Palaeontology and archaeology
☐	☒	Animals and other organisms
☒	☐	Human research participants
☒	☐	Clinical data
☒	☐	Dual use research of concern

Methods

n/a	Involved in the study	
☒	☐	ChIP-seq
☒	☐	Flow cytometry
☒	☐	MRI-based neuroimaging

Antibodies

Antibodies used	(1)Anti-PCNAwas obtained from ABclonal (Wuhan, China).<br>(2)Anti-β-actin was purchased from Cell Signaling Technology (Beverly, MA).<br>(3)Anti-claudin-1 was obtained from Abcam (Cambridge, MA).
Validation	Only commercially available antibodies were used. All antibodies used have been tested and validated by the suppliers and other researchers.

Animals and other organisms

Policy information about studies involving animals; ARRIVE guidelines recommended for reporting animal research

Laboratory animals	C57BL/6J background mice
Wild animals	No wild animals used in this study.
Field-collected samples	No field-collected samples was used.
Ethics oversight	All experiments were conducted in strict accordance with the Guide for the Care and Use of Laboratory Animals prepared by the National Academy of Sciences (NIH publication 86-23, revised 1985). Experimental protocols were approved by the Animal Care and Use Committee of Peking University.

Note that full information on the approval of the study protocol must also be provided in the manuscript.
